# Optimization of murine retinal mitochondrial injury model

**DOI:** 10.1016/j.mex.2022.101701

**Published:** 2022-04-16

**Authors:** Xiaopeng Zhou, Gengjing Fang, Liping Zhang

**Affiliations:** The Molecular Neuropharmacology Laboratory and the Eye-Brain Research Center, The State Key Laboratory of Ophthalmology, Optometry and Vision Science, School of Optometry and Ophthalmology, Wenzhou Medical University, Wenzhou 325000, China

**Keywords:** Retinal injury, Mitochondrial injury, CCCP, Opa1, A_2A_ receptor

## Abstract

The retinal mitochondrial injury model in rat has been developed using the mitochondrial oxidative phosphorylation uncoupler, carbonylcyanide m-chlorophenyl hydrazine (CCCP). However, the CCCP-induced murine retinal mitochondrial injury model has not been reported. Here, the optimized conditions for the murine retinal mitochondrial injury model were established by intravitreal injection of different doses of CCCP (0, 2.5, 5, 7.5, 10, 12.5, 15 μg). Indeed, it has been reported that CCCP induces Opa1 cleavage and phosphorylation of ERK in cultured cells and rat retinas. Thus, we measured phosphorylated (p) -Erk and L/S-Opa1 following CCCP-induced retinal injury. Meanwhile, KW6002 (A_2A_ receptor antagonist) pretreatment inhibited retinal injury induced by CCCP at 10 and 15 μg doses differently. Intravitreal injection of 10 μg doses of CCCP can induce apoptosis of retinal ganglion cells and decrease of retinal thickness, but intravitreal injection of 15 μg doses of CCCP is the appropriate dose to study the protective effect of A_2A_ receptor.

(1) Dose dependent effects of intravitreal injection of CCCP on the levels of L/S-Opa1 and p-Erk;

(2) A_2A_ receptor antagonist (KW6002) only inhibited the apoptosis of ganglion cells, but did not affect the thickness of retina with 10µg dosage of CCCP intravitreal injection;

(3) A_2A_ receptor antagonist (KW6002) inhibited the apoptosis of ganglion cells and increased the thickness of retina with 15µg dosage of CCCP intravitreal injection.

## Graphical abstract

**Figure:**
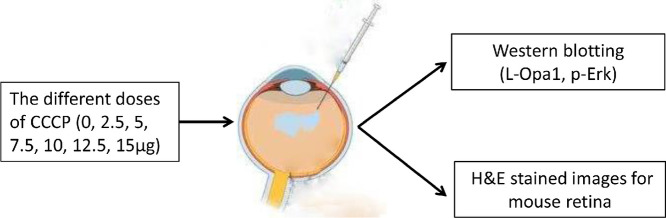

**Specifications table**

**Table:** 

Subject Area;	
More specific subject area;	*Retinal injury*
Method name;	*murine retinal mitochondrial injury model*
Name and reference of original method;	*Sun et al.*[7]*, Restoration of Opa1-long isoform inhibits retinal injury-induced neurodegeneration, J Mol Med (Berl), 94(3), 335-346.*
Resource availability;	*The Molecular Neuropharmacology Laboratory and the Eye-Brain Research Center, The State Key Laboratory of Ophthalmology, Optometry and Vision Science, School of Optometry and Ophthalmology, Wenzhou Medical University*

## Background

Carbonyl cyanide 3-chlorophenylhydrazone(CCCP), the mitochondrial oxidative phosphorylation uncoupler, disruptes the mitochondrial membrane potential to trigger various stress pathways and induces the production of reactive oxygen species (ROS) [1], [2], [3], [4]. Accumulating evidence shows that oxidative stress and mitochondrial dysfunction are aggravating factors in neurodegenerative disorders [5], [6]. Intravitreal injection of CCCP was sufficient to lead to retinal neurodegeneration and mimicked molecular characteristics and tissue injury phenotypes caused by retinal ischemia–reperfusion injury in rat [7]. The method described in this research aims to establish optimized conditions for the mitochondrial injury of murine retina model.

## Method details

### Mice and animal care

Adult (8–10 weeks old) C57B6/J mice with the weight of 20–22 g, were purchased from SPF (Beijing) Biotechnology Co., Ltd. Animal care and use were approved by the Institutional Ethics Committee for Animal Use in Research and Education at Wenzhou Medical University, China. The mice were maintained with a 12/12 photoperiod (light on at 8 AM).

### The mitochondrial injury of murine  retinal model

**Fig. 1:**
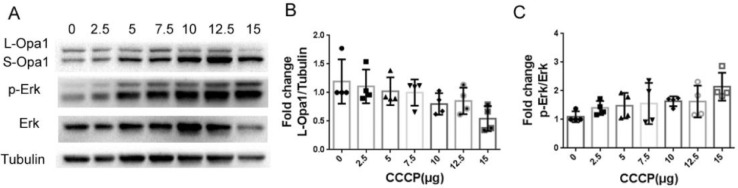
Dose dependent effects of intravitreal injection of CCCP on the levels of Opa1 and p-Erk. (A) Representative western blotting of the levels of Opa1, p-Erk, Erk and tubulin for the indicated dosages of CCCP. (B) Quantitative changes of L-Opa1 for the indicated dosages of CCCP (One way ANOVA, *P* < 0.05). (C) Quantitative changes of P-Erk/Erk for the indicated dosages of CCCP (One way ANOVA, *P* < 0.05).

Adult (8–10 weeks old) C57B6/J mice were anesthetized with pentobarbital (i.p. 60 mg/kg). Carbonyl cyanide 3-chlorophenylhydrazone (CCCP, CAS No.: 555-60-2, MCE) was diluted to 1.5 µg/µL,12.5 µg/µL,10 µg/µL, 7.5 µg/µL, 5 µg/µL,2.5 µg/µL with dimethyl sulfoxide (DMSO, D2650, Sigma). A thirty-Gauge needle (Hamilton, USA) was inserted using a Hamilton microinjector (Hamilton Company, USA) toward the optic nerve 1 mm outside of the limbus under a microscope. The CCCP or DMSO were slowly injected after the needle tip was detected in the pupil area. A volume containing 1 μL of undiluted DMSO was intravitreally injected into the right eye, whereas a volume containing 1 μL of 1.5 µg/µL,12.5 µg/µL,10 µg/µL, 7.5 µg/µL, 5 µg/µL,2.5 µg/µL CCCP were intravitreally injected into the left eye. After 24 h, the mice were harvested, and the retinas were collected to extract total protein for western blotting. After 48 h, the mice were harvested, and Fresh eyeballs were collected for paraffin section and H&E staining (Fig. 1).

### Western blotting

Freshly isolated retinas were soniccated in an ice-cold RIPA buffer (Beyotime, China), and protein concentrations were quantitated. Thirty to forty-five μg of protein per sample was separated by SDS-PAGE and then transferred onto PVDF membranes (Millipore, USA) for immunodetection. The membranes were incubated in the following antibodies: OPA1 (1:1000; Cell Signaling Technology; 67589), ERK1/2 (1:1000; proteintech; 16443-1-AP)

### KW6002 treatment

KW6002 was prepared freshly in 15% DMSO, 15% Castrol oil, and 70% H2O at a final concentration of 0.3 mg/mL, as described previously [8].

### H&E staining

**Fig. 2:**
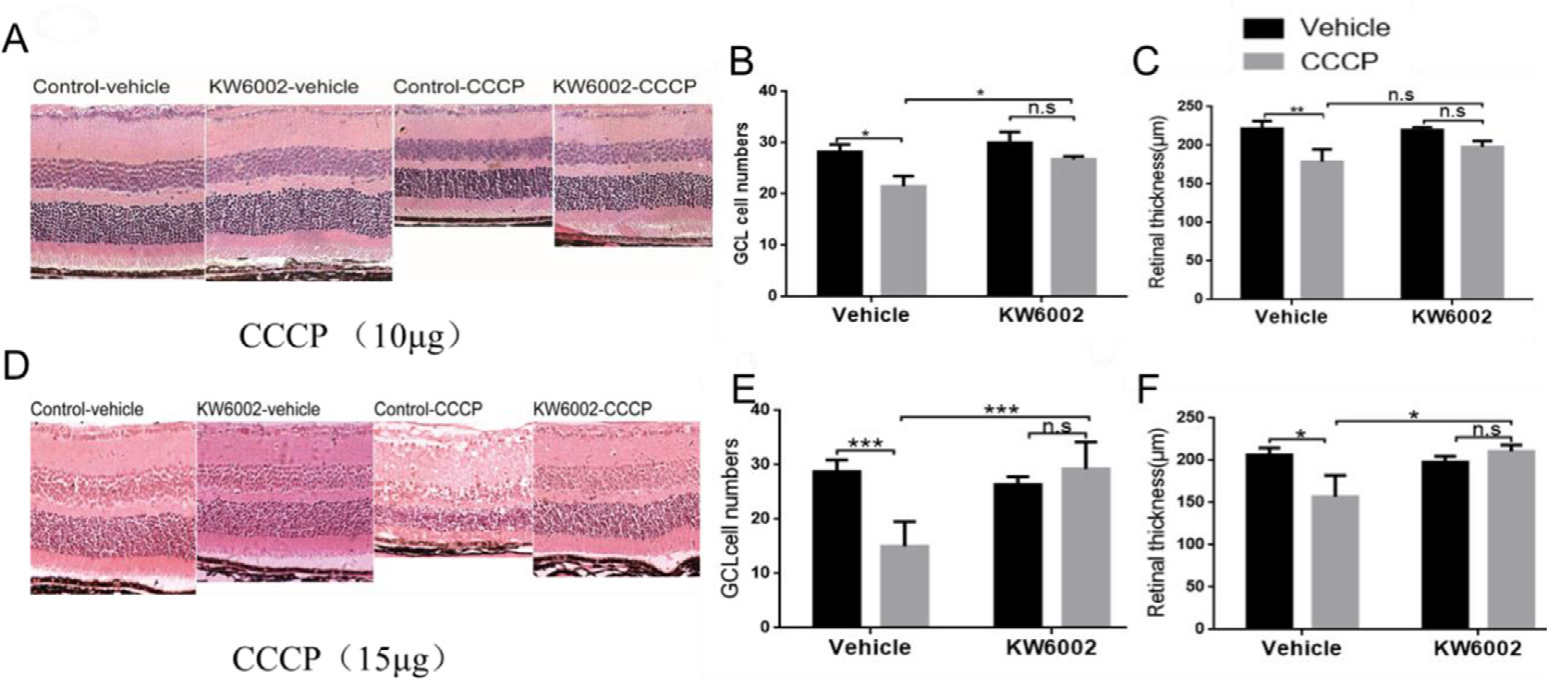
Pretreatment with the A_2A_ receptor antagonist KW6002 (10 µg/15 µg) reduces CCCP-induced retinal injury. (A–C) Representative H&E stained images and quantitation of changes in retinal thickness, IPL thickness and the RGC numbers in the indicated experimental groups (*n* = 5). (D–F) Same as A–C, but for the dosage of 15 µg CCCP. * = *P* < 0.05; ** = *P* < 0.01; *** = *P* < 0.001, One way ANOVA, followed by post-hoc comparison with LSD test.

Fresh eyeballs were harvested and soaked overnight in 4% paraformaldehyde at 4°C, then dehydrated in an ascending series of ethanol, and equilibrated with xylene, followed by embedding in paraffin and sectioning into 5 μm slices. Then, the samples were dewaxed with xylene and a descending series of ethanol. Continued sections were stained with both hematoxylin and eosin (H & E). The samples were observed and photographed under a confocal microscope (Leica, Germany) (Fig. 2).

## Declaration of Competing Interest

The authors have declared no conflict of interest.
